# Snapchat-Based Structured Education Reduces Kinesiophobia and Improves Psychological Readiness and Perceived Knee Function Following Anterior Cruciate Ligament Reconstruction: A Quasi-Experimental Study

**DOI:** 10.3390/jcm15093385

**Published:** 2026-04-29

**Authors:** Abdullah H. AlMuhaya, Thamer Alshahrani, Abdulsalam Alshammari, Salman Alsudairi, Mai Aldera, Dalia M. Alimam

**Affiliations:** 1 Joint Clinics, Medical Rehabilitation Center, Riyadh 13515, Saudi Arabia; ithamer77@gmail.com (T.A.); ptabdussalam.n@gmail.com (A.A.); smalsudairi@gmail.com (S.A.); 2 Department of Health Rehabilitation Sciences, College of Applied Medical Sciences, King Saud University, Riyadh 11451, Saudi Arabia; maldera@ksu.edu.sa (M.A.); dalimam@ksu.edu.sa (D.M.A.)

**Keywords:** psychological readiness, social media, digital health, fear avoidance, return to sport, rehabilitation

## Abstract

**Background/Objectives:** Psychological barriers, particularly kinesiophobia and diminished psychological readiness, represent critical yet undertreated obstacles to a successful return to sport following anterior cruciate ligament reconstruction (ACLR). Scalable, preference-aligned educational interventions capable of addressing these barriers during early rehabilitation are lacking. We aimed to evaluate the effectiveness of structured educational content delivered via Snapchat, as an adjunct to standard ACLR rehabilitation, in reducing kinesiophobia (primary outcome) and improving psychological readiness and perceived knee function (secondary outcomes). **Methods:** A total of 120 adults with clinically elevated kinesiophobia (TSK-17 > 37) undergoing post-operative ACLR rehabilitation were enrolled in a quasi-experimental, two-arm study with non-randomized allocation at the clinic-branch level at two branches of the same sports rehabilitation clinic (Joint Clinics, Riyadh, Saudi Arabia). Branch allocation assigned 60 participants to each group (intervention and control). The intervention group received 12 weekly structured educational videos via Snapchat alongside standard rehabilitation; the control group received standard rehabilitation alongside general ACLR information videos via Snapchat. TSK-17, ACL-RSI, and IKDC were assessed at baseline and at 12 weeks. Primary analysis used ANCOVA covarying baseline scores, complemented by mixed repeated measures ANOVA and intent-to-treat analysis. **Results:** Both groups improved across all outcomes; the intervention group demonstrated significantly greater gains. ANCOVA revealed significant between-group differences favoring the intervention for TSK-17 (adjusted mean difference = −2.82; d = 0.54; *p* < 0.001; d represents Cohen’s d calculated from adjusted mean differences and pooled SD), ACL-RSI (+8.06; d = 0.77; *p* < 0.001), and IKDC (+8.90; d = 0.54; *p* = 0.002). Mean video completion was 82.8% among intervention participants. Intent-to-treat analyses using Multiple Imputation confirmed all findings. **Conclusions:** Snapchat-based structured education was associated with improvements in kinesiophobia, psychological readiness, and perceived knee function among the 102 analyzed participants (control *n* = 52; intervention *n* = 50) of the 120 enrolled. High engagement supports preference-based digital delivery as a scalable adjunct to standard rehabilitation.

## 1. Introduction

### Background

Anterior cruciate ligament reconstruction (ACLR) is among the most performed orthopedic procedures worldwide, yet return-to-sport (RTS) outcomes remain suboptimal. Only 55–65% of patients return to their pre-injury sport level [1,2], fewer than 50% regain pre-injury performance [3], and re-injury risk within the first two years is up to six times that of uninjured individuals, with rates of 23–29% and a contralateral risk of up to 20% [4,5].

Psychological factors are increasingly recognized as critical contributors to RTS outcomes [6,7]. Kinesiophobia is consistently identified as a major barrier to successful RTS, associated with impaired neuromuscular performance, delayed functional recovery, reduced self-efficacy, and increased secondary ACL injury risk [8,9]. Importantly, kinesiophobia and neuromechanical function are reciprocally linked: elevated fear of movement has been associated with altered lower-limb biomechanics during functional tasks, including reduced knee flexion excursion, stiff-knee landing patterns, and greater inter-limb asymmetry, which in turn may reinforce fear-avoidance beliefs and delay unrestricted return to activity [10]. Psychological readiness, encompassing confidence, emotional control, and mental preparedness, features among the strongest predictors of RTS, often surpassing objective physical measures [6,11]. Despite this evidence, contemporary rehabilitation protocols continue to prioritize biomechanical criteria, with limited structured psychological integration [12,13].

Kinesiophobia typically peaks during early rehabilitation (0–3 months post-surgery), when beliefs remain modifiable and maladaptive fear-avoidance patterns have not yet become entrenched [14,15,16]. Phase-specific longitudinal evidence from a Saudi ACLR cohort confirms that the most pronounced fear reduction occurs mid-rehabilitation, with stabilization in later phases [16]. Educational interventions offer a practical, scalable strategy to address these barriers by correcting misconceptions, normalizing pain-related concerns, and promoting graded movement exposure [12,17]. However, most existing psychosocial interventions require face-to-face delivery or specialized psychological input, limiting scalability in routine clinical settings [12,18]. Social media platforms offer a novel medium for flexible, cost-effective educational delivery. Short-form video platforms align with established principles of micro-learning and short-form visual reinforcement in medical education, including bite-sized content delivery, spaced repetition, and multimodal encoding, which have been shown to improve knowledge retention and behavior change compared with traditional didactic approaches [19]. In Saudi Arabian contexts, Snapchat has emerged as a highly preferred platform for health-related content, with its short-form visual format facilitating engagement and message retention [20,21].

To our knowledge, no study thus far has examined the effectiveness of structured ACLR educational content delivered via social media on psychological and functional rehabilitation outcomes. Structured interventions targeting kinesiophobia and psychological readiness during early rehabilitation using validated outcome measures have not been tested in Arabic-speaking ACLR populations. Preference-aligned delivery may maximize engagement and participation, as a key determinant of intervention effectiveness [22,23].

This quasi-experimental study evaluated whether Snapchat-based structured educational content, as an adjunct to standard ACLR rehabilitation, could reduce kinesiophobia (primary outcome) and improve psychological readiness and perceived knee function (secondary outcomes). It was hypothesized that the intervention group would experience greater reductions in kinesiophobia and superior improvements in psychological readiness and perceived knee function compared with the control group.

## 2. Methodology

### 2.1. Study Design

This quasi-experimental study with non-randomized branch-level allocation evaluated Snapchat-based structured educational content as an adjunct to standard ACLR rehabilitation over 12 weeks. Outcome measures, assessment timing, and data collection procedures were identical across both groups.

### 2.2. Setting and Recruitment

Recruitment was conducted at two branches of the same sports rehabilitation service (Joint Clinics, Riyadh, Saudi Arabia) between May 2025 and January 2026. Allocation was non-randomized at the clinic-branch (cluster) level, with Branch 1 assigned as control and Branch 2 as intervention. This design was selected because the intervention was delivered via Snapchat (Snap Inc., Santa Monica, CA, USA), a peer-connected platform on which within-site individual allocation would have permitted contamination through patient-to-patient content sharing, and because branch-level allocation was necessary to preserve therapist blinding. The trade-offs inherent to this design, including residual cluster-level confounding, are addressed in Section 4.6. Both branches operate under a unified, phase-based, criteria-driven ACLR rehabilitation protocol with identical staffing models, training procedures, and electronic medical records.

### 2.3. Participants and Eligibility Criteria

Participants were adults undergoing post-operative ACLR rehabilitation with clinically elevated kinesiophobia (TSK-17 > 37; [24,25]). The Arabic TSK-17 has been validated for use in ACLR rehabilitation [25], and a score above 37 has been applied as a clinically relevant threshold in post-ACLR contexts [25], although this cutoff was originally derived from chronic low back pain populations [24], and has not, to our knowledge, been independently validated in ACLR populations. The additional inclusion criteria for all participants were age ≥ 18 years, willingness to provide informed consent, ability to complete Arabic-language questionnaires, active enrollment at a participating branch, and an active Snapchat account. Exclusion criteria included prior ACL revision surgery, concurrent major lower-limb injury, neurological or medical conditions independently affecting rehabilitation, and non-compliance with study procedures. No restriction was placed on the rehabilitation phase at enrollment. Participants were recruited across Phases 1 and 2 (0–3 months post-surgery), consistent with evidence that kinesiophobia is highest and most modifiable during early rehabilitation [14,15,16]. The TSK-17 > 37 threshold served as the primary eligibility criterion, ensuring that enrolled participants had clinically elevated fear regardless of phase, and maximizing recruitment feasibility within the targeted early rehabilitation window.

### 2.4. Outcome Measures

Three validated Arabic patient-reported outcome measures were administered at baseline and at the 12-week follow-up. The primary outcome was kinesiophobia, assessed using the TSK-17 ([24]; scored 17–68; higher = greater kinesiophobia; ICC = 0.93; α = 0.90; [25]). Secondary outcomes were psychological readiness via the ACL-RSI ([26]; 0–100; higher = greater readiness; α = 0.93; ICC = 0.93; [27]) and perceived knee function via the IKDC ([28]; 0–100; higher = better function; α = 0.92; ICC = 0.97; [29]). In addition to outcome measures, demographic and clinical data, including age, height, weight, BMI, dominant leg, injured leg, graft type, rehabilitation phase, and number of physical therapy sessions completed, were collected at baseline.

### 2.5. Intervention

**Figure A1:**
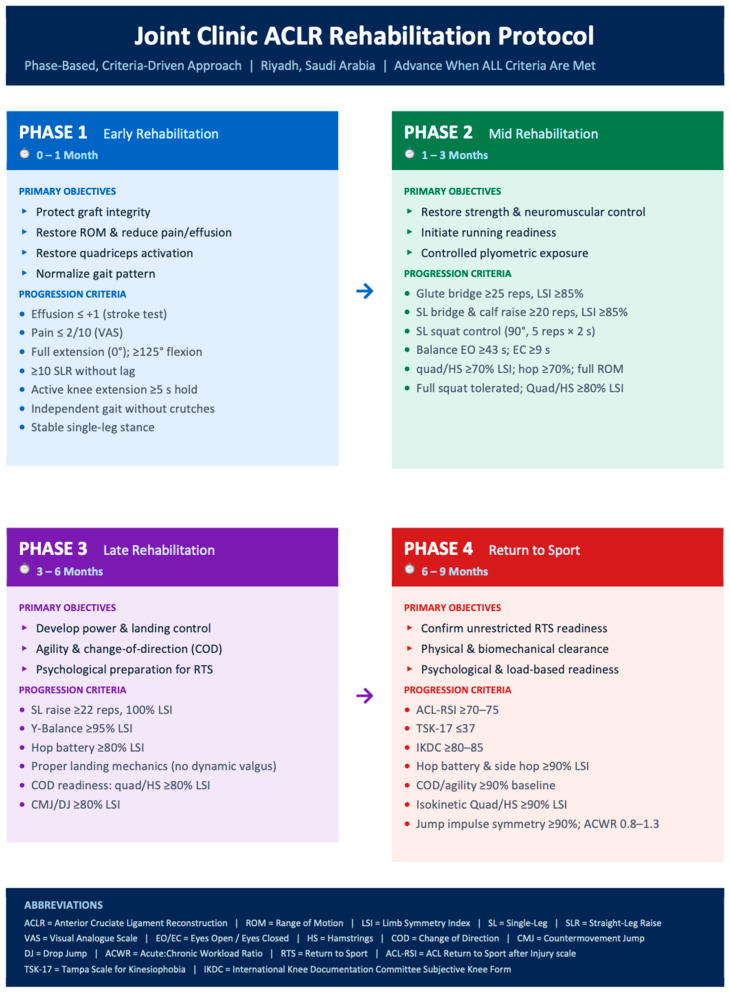
Joint Clinics ACLR rehabilitation protocol: a phase-based, criteria-driven approach (Joint Clinics, Riyadh, Saudi Arabia). Participants advance to the next phase only when all progression criteria are met.

**Figure A2:**
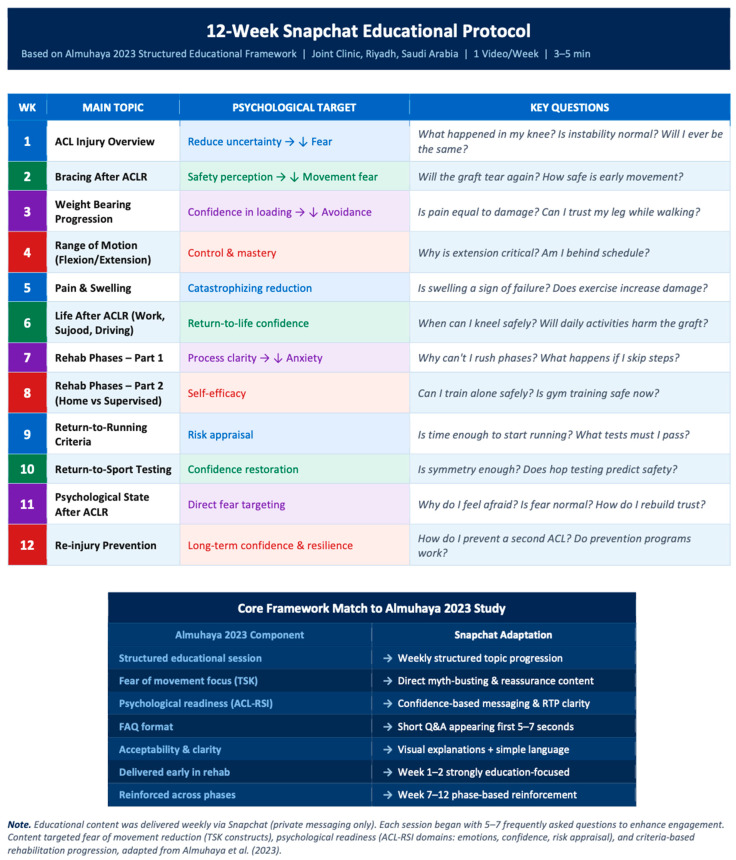
The 12-week Snapchat educational protocol, adapted from Almuhaya et al. [17]. Note: Arrows (→) indicate directional relationships between topics and psychological targets; downward arrows (↓) indicate intended reductions in the associated construct.

All participants received the standardized ACL rehabilitation protocol uniformly across both branches (Figure A1). The intervention group additionally received a structured 12-week educational program via Snapchat (Figure A2). The control group received general ACLR information videos via Snapchat, matched in frequency and platform to the intervention group but without structured psychological education. The first video was delivered within 48 h of baseline, and one video (3–5 min) was sent weekly via individual private messaging to all participants. All videos for both groups were delivered by a single research team member (A.A.), who was the only individual with knowledge of group allocation. Intervention content was adapted from a validated educational framework [17], and addressed fear of re-injury, expectation management, graded exposure, and RTS readiness in a manner that was grounded in fear-avoidance theory. Engagement was recorded via Snapchat analytics (videos viewed; program completion defined as ≥9 of 12 videos to ensure sufficient exposure to intervention content while allowing for realistic attrition).

### 2.6. Bias Control and Sample Size

The therapists performing treatment, participants, the outcome assessor, and the data analyst were all blinded to group allocation. Allocation was known only to the research team member responsible for video delivery (A.A.). Participant blinding was achieved through delivery of Snapchat videos to both groups, with content differing in structure and specificity but not in platform or frequency. Participants were not informed of specific study hypotheses. An a priori power analysis using ANCOVA (G*Power 3.1.9.7, Heinrich-Heine-Universität, Düsseldorf, Germany; *F* tests: ANCOVA fixed effects; [30]), based on a meta-analytic Cohen’s d = 0.56 [31] converted to Cohen’s f = 0.28 (f = d/2; [32]), with 2 groups, 1 covariate, and numerator df = 1, indicated a minimum sample of 103 participants (α = 0.05, power = 0.80). The recruitment target was set at 120 (60 per group), providing a 15% attrition buffer. The analysis was conducted at the individual level per standard practice for ANCOVA; a cluster design effect could not be incorporated because the intra-cluster correlation coefficient is not estimable with only two clusters, a constraint discussed further in the limitations (Section 4.6).

### 2.7. Statistical Analysis

Statistical analyses were conducted using SPSS version 31.0 (IBM Corp., Armonk, NY, USA). Graphical illustrations and figures were generated using Numiqo (https://numiqo.com, accessed on 11 March 2026). Baseline comparability was assessed using independent samples *t*-tests and chi-square tests. The primary analysis was ANCOVA, with the 12-week score as the dependent variable, group as the fixed factor, and the corresponding baseline score as the covariate [33]. No additional covariates were included, given confirmed baseline equivalence across all demographic and clinical variables. ANCOVA was complemented by 2 × 2 mixed repeated measures ANOVA to characterize change trajectories over time and enable visualization of group differences across assessment points. Effect sizes were reported as partial eta squared (η^2^p; small = 0.01, medium = 0.06, large = 0.14) and Cohen’s d (small = 0.2, medium = 0.5, large = 0.8; [32]). Statistical significance was set at *p* < 0.05. Intent-to-treat analyses used Multiple Imputation (MI) to handle missing 12-week outcome data. Fifty imputed datasets (M = 50) were generated via multivariate imputation by chained equations (MICE), incorporating all three outcome measures (TSK-17, ACL-RSI, IKDC), baseline covariates, and auxiliary variables, with 20 burn-in iterations preceding the final imputations. The number of imputations was set consistent with published guidance that M should equal or exceed the percentage of missing data [34,35]. ANCOVA models were fitted within each imputed dataset and pooled using Rubin’s rules, with Barnard–Rubin adjusted degrees of freedom. Last Observation Carried Forward (LOCF) imputation was additionally conducted as a sensitivity analysis.

### 2.8. Ethical Approval and Registration

Ethical approval was granted by the Research Ethics Committee, Prince Sattam bin Abdulaziz University (RHPT/025/006; 4 May 2025). The study was prospectively registered with the Research Registry (researchregistry11773). All participants provided written informed consent. Data were anonymized, stored on encrypted systems, and retained for five years per Saudi data protection regulations. This study adheres to the CONSORT extension for non-randomized trials [36].

## 3. Results

### 3.1. Participant Flow and Attrition

**Figure 1:**
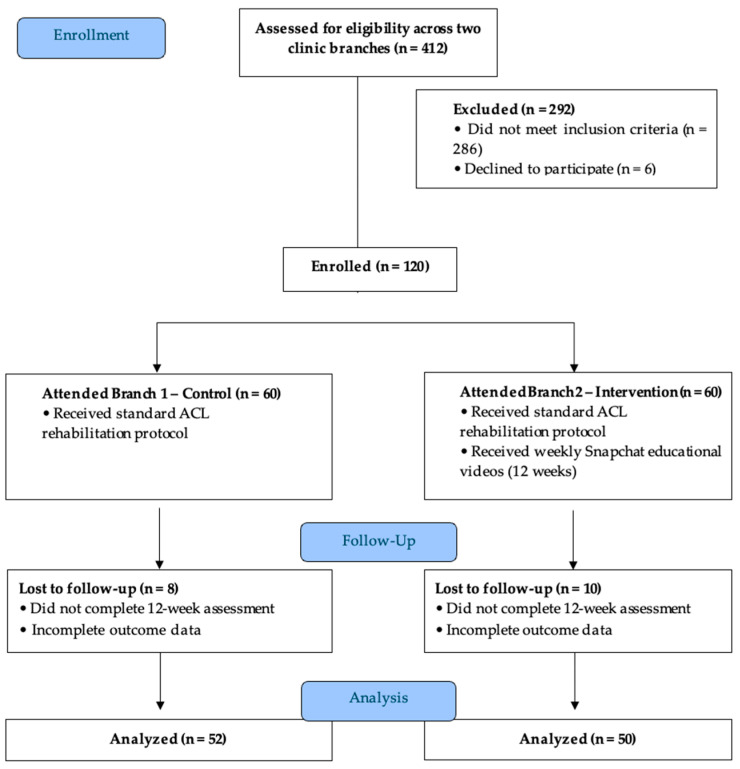
Flow diagram of participant recruitment, branch allocation, follow-up, and analysis.

Of the 412 individuals screened, 292 were excluded (286 did not meet inclusion criteria; 6 declined). A total of 120 participants were enrolled (60 per group). At 12 weeks, 8 control (13.3%) and 10 intervention (16.7%) participants were lost to follow-up; attrition rates did not differ significantly (χ^2^(1) = 0.261, *p* = 0.609). The per-protocol sample comprised 52 control and 50 intervention participants (*n* = 102; 85.0% retention). See Figure 1 for a visual representation of participant recruitment, branch allocation, follow-up, and analysis. Baseline characteristics of participants lost to follow-up were: TSK-17 = 41.3 ± 4.9, ACL-RSI = 39.4 ± 4.9, and IKDC = 51.6 ± 9.7. For comparison, those of completers were as follows: TSK-17 = 43.5 ± 4.1, ACL-RSI = 40.1 ± 5.5, and IKDC = 47.3 ± 12.7. Dropout timing for control participants (*n* = 8) could not be determined, as video engagement was not tracked for the control group.

### 3.2. Baseline Characteristics

No statistically significant between-group differences were observed for any of the baseline variables (all *p* > 0.05), confirming comparability across age, anthropometrics, rehabilitation phase, physical therapy sessions, graft type, limb dominance, and all three outcome scores (TSK-17: *p* = 0.541; ACL-RSI: *p* = 0.148; IKDC: *p* = 0.681; Table 1).

### 3.3. Outcome Measures at Baseline and 12 Weeks

**Figure 2:**
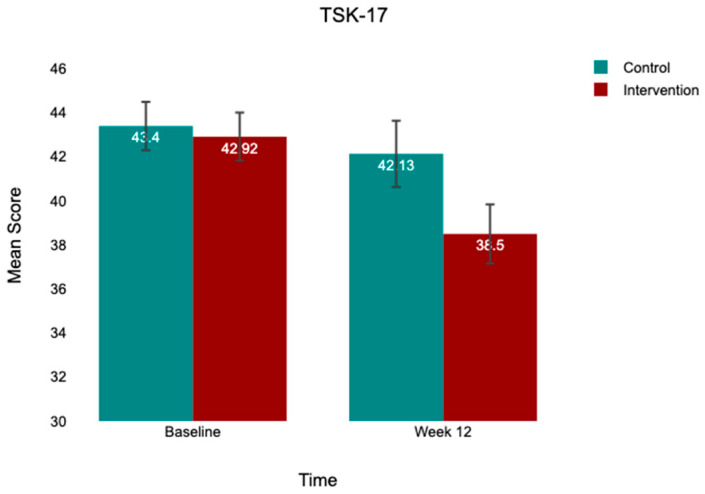
Mean TSK-17 scores at baseline and week 12 by group. Note: Bar heights = group means; error bars = 95% CI. Control (*n* = 52); intervention (*n* = 50). Between-group difference at week 12 is statistically significant after controlling for baseline (*p* < 0.001).

**Figure 3:**
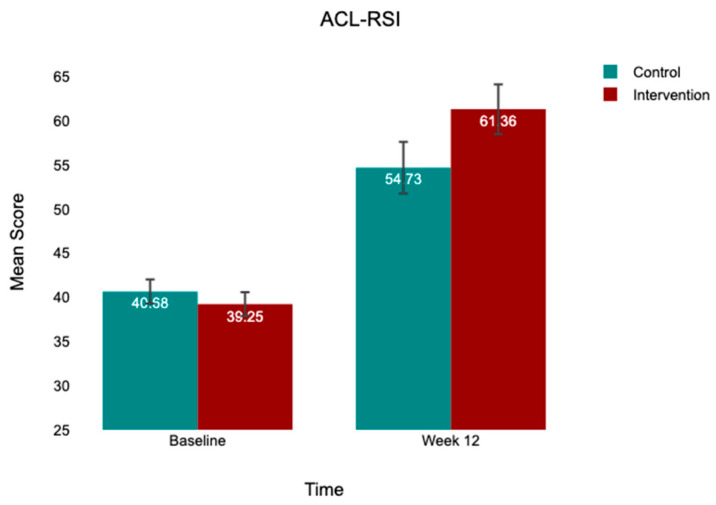
Mean ACL-RSI scores at baseline and week 12 by group. Note: Bar heights = group means; error bars = 95% CI. Control (*n* = 52); intervention (*n* = 50). Between-group difference at week 12 is statistically significant after controlling for baseline (*p* < 0.001).

**Figure 4:**
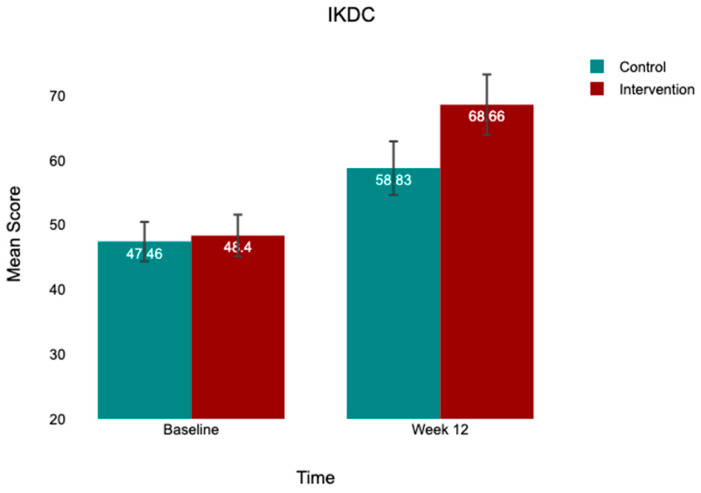
Mean IKDC scores at baseline and week 12 by group. Note: Bar heights = group means; error bars = 95% CI. Control (*n* = 52); intervention (*n* = 50). Between-group difference at week 12 is statistically significant after controlling for baseline (*p* = 0.002).

At baseline, both groups exhibited comparable kinesiophobia (TSK-17: control, 43.96 ± 3.96; intervention, 43.00 ± 4.27), low-to-moderate psychological readiness (ACL-RSI: control, 40.98 ± 5.40; intervention, 39.10 ± 5.53), and moderate knee function (IKDC: control, 46.48 ± 12.23; intervention, 48.09 ± 13.31). At 12 weeks, both groups improved across all outcomes, with the intervention group demonstrating substantially greater gains (Table 2; Figure 2, Figure 3 and Figure 4).

### 3.4. Between-Group Comparisons

Between-group differences were examined using two complementary analyses: 2 × 2 mixed repeated measures ANOVA and ANCOVA covarying baseline scores. Homogeneity of variance was confirmed for TSK-17 (*p* = 0.708) and ACL-RSI (*p* = 0.796). A significant violation was observed for IKDC (*F*(1,100) = 15.50, *p* < 0.001); accordingly, the IKDC ANCOVA was re-estimated using heteroscedasticity-consistent (HC3) robust standard errors, with a non-parametric sensitivity analysis (Quade’s rank ANCOVA) conducted to corroborate findings.

### 3.5. Repeated Measures ANOVA

**Figure 5:**
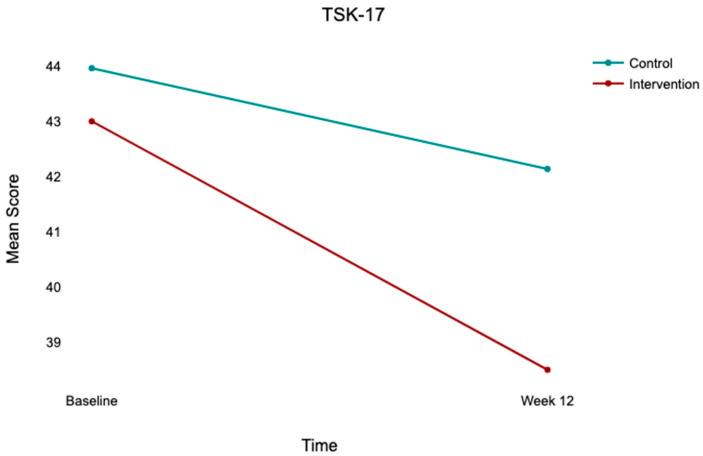
Time × group interaction for TSK-17 scores from baseline to week 12. Note: Lines connect group means at each time point. Control (*n* = 52); intervention (*n* = 50). There is significant time × group interaction (*F*(1,100) = 11.78, *p* < 0.001, η^2^p = 0.105), indicating greater reduction in kinesiophobia in the intervention group.

**Figure 6:**
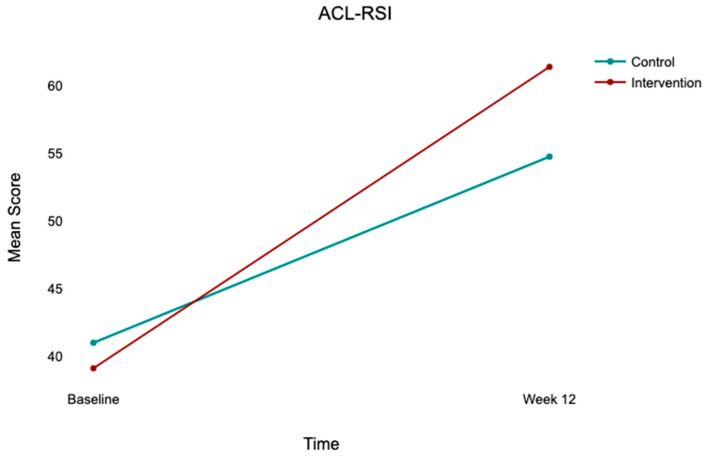
Time × group interaction for ACL-RSI scores from baseline to week 12. Note: Lines connect group means at each time point. Control (*n* = 52); intervention (*n* = 50). There is significant time × group interaction (*F*(1,100) = 19.54, *p* < 0.001, η^2^p = 0.163), indicating greater improvement in psychological readiness in the intervention group.

**Figure 7:**
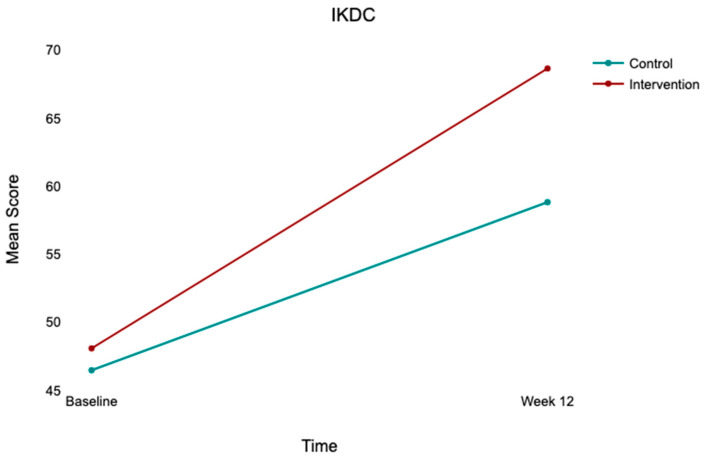
Time × group interaction for IKDC scores from baseline to week 12. Note: Lines connect group means at each time point. Control (*n* = 52); intervention (*n* = 50). There is significant time × group interaction (*F*(1,100) = 7.317, *p* = 0.008, η^2^p = 0.068), indicating greater improvement in knee function in the intervention group.

Significant main effects of time were observed across all outcomes: TSK-17 (*F*(1,100) = 66.00, *p* < 0.001, η^2^p = 0.398), ACL-RSI (*F*(1,100) = 349.95, *p* < 0.001, η^2^p = 0.778), and IKDC (*F*(1,100) = 117.38, *p* < 0.001, η^2^p = 0.540). Significant time × group interactions were observed for all three outcomes (Table 3; Figure 5, Figure 6 and Figure 7), indicating that improvement trajectories differed between groups. Post hoc comparisons confirmed there were no significant between-group differences at baseline (all *p* > 0.05), but that there were significant differences at week 12 for TSK-17 (mean difference = 3.63, *p* = 0.004), ACL-RSI (mean difference = −6.63, *p* = 0.011), and IKDC (mean difference = −9.83, *p* = 0.016), all favoring the intervention group.

### 3.6. ANCOVA

After adjusting for baseline scores, the intervention group demonstrated significantly better outcomes on all three measures (Table 4): TSK-17 (adjusted mean 38.91 vs. 41.74; mean difference = −2.82, *F*(1,99) = 13.19, *p* < 0.001, η^2^p = 0.118, *d* = 0.54, moderate); ACL-RSI (62.09 vs. 54.03; +8.06, *F*(1,99) = 17.17, *p* < 0.001, η^2^p = 0.148, *d* = 0.77, moderate-to-large); and IKDC (68.19 vs. 59.29; +8.90, *F*(1,99) = 9.670, *p* = 0.002, η^2^p = 0.089, *d* = 0.54, moderate). Robust SE re-estimation for IKDC confirmed the effect (adjusted mean difference = +8.90, robust SE = 2.98, 95% CI [2.98, 14.82], *p* = 0.003); Quade’s rank ANCOVA yielded a convergent result (*p* = 0.002).

### 3.7. Intent-to-Treat Analysis

ITT analyses (*n* = 120) using Multiple Imputation (MI) (M = 50) confirmed findings across all outcomes (Table 5). Pooled ANCOVA using Rubin’s rules yielded significant between-group differences favoring the intervention for all three outcomes (all *p* ≤ 0.003; Table 5). Fractions of missing information ranged from 0.10 to 0.15, indicating low impact of missingness on pooled estimates. Last Observation Carried Forward (LOCF)-based sensitivity analysis produced convergent results (Table 6), supporting robustness of findings for the choice of missing-data method. The convergence of per-protocol, MI-based ITT, and LOCF sensitivity findings strengthens confidence in intervention effects.

### 3.8. Intervention Engagement and Adherence

Engagement data were available for all 60 intervention participants. The mean number of videos viewed was 9.93 ± 1.46 (range: 8–12), corresponding to a mean completion rate of 82.8%. All participants viewed a minimum of 8 videos; 47 (78.3%) met the ≥75% completion threshold, and 13 (21.7%) viewed all 12. Among the 10 participants lost to follow-up, the mean number of videos completed was 10.50; specifically, six had viewed 10 videos and four had viewed 11–12 videos at the time of dropout, including one participant who viewed all 12 videos but did not attend the assessment at week 12. Video-view data were captured automatically via Snapchat’s private messaging analytics, independent of study follow-up status, which allowed engagement to be recorded even for participants who did not complete the 12-week assessment.

## 4. Discussion

This study provides evidence that structured educational content delivered via Snapchat as an adjunct to standard ACLR rehabilitation is associated with statistically significant improvements across all three outcomes, with effect sizes ranging from moderate to moderate-to-large. High engagement and convergence across analytical approaches support the potential of preference-based digital education as a meaningful adjunct to ACLR rehabilitation.

### 4.1. Theoretical Basis

The observed improvements align with the fear-avoidance model, which posits that catastrophic misinterpretation of pain leads to fear-driven movement avoidance and delayed recovery [37,38]. The intervention targeted multiple components of this cycle: expectation management reframed post-surgical symptoms as expected recovery phenomena, consistent with cognitive reappraisal approaches that reduce threat perception in musculoskeletal populations [39]; uncertainty reduction provided clear information about rehabilitation progression, potentially decreasing ambiguity-driven anxiety [40]; and educational content normalized fear while distinguishing pain from harm. This approach contrasts with purely biomechanical rehabilitation models that assume that psychological recovery occurs automatically as physical function improves, a premise challenged by evidence of persistent psychological barriers among patients meeting objective RTS criteria [2,8].

### 4.2. Comparison with the Existing Literature

The observed effects exceed those of previous ACLR psychosocial intervention trials. Coronado et al. [12] reported small-to-moderate effects on fear reduction, attributing limited efficacy to late intervention timing (median four months post-surgery) and clinic-based delivery. The current study intervened during the earliest rehabilitation phases via asynchronous delivery, achieving substantially higher completion rates. Nwachukwu et al. [18] demonstrated that low psychological readiness predicted reduced RTS rates but did not test interventions to enhance it; the current study extends this work by demonstrating that structured education can meaningfully improve readiness during active rehabilitation. Single-session group education has shown minimal psychological change [41], likely reflecting insufficient exposure; weekly sequencing over 12 weeks provided repeated, phase-matched reinforcement aligned with evolving rehabilitation challenges.

The clinical meaningfulness of between-group differences warrants interpretation against available benchmarks. The TSK-17 difference of 2.82 points (*d* = 0.54) should be interpreted cautiously, as no validated MCID exists for the TSK-17 in ACLR populations. The commonly referenced 5.5-point threshold was derived from chronic low back pain [42] and may not translate directly to post-operative cohorts. The ACL-RSI improvement of 8.06 points (*d* = 0.77) exceeded the SEM of 4.42 points [27], indicating genuine improvement beyond measurement error, though the smallest detectable difference of 12.22 points was not exceeded at the individual level. The IKDC improvement of 8.90 points (*d* = 0.54) approached the true change threshold of 9.0 points [28] and exceeded the SDC of 7.2 points [43], indicating that the observed improvement is unlikely to reflect measurement error.

### 4.3. Digital Delivery and Engagement

The 82.8% completion rate contrasts markedly with typical digital health interventions reporting substantially lower rates [22,23] and likely reflects preference-based platform selection [44]. Snapchat’s short-form format aligns with cognitive load principles by presenting information in digestible segments [45], while asynchronous access accommodates variable rehabilitation schedules. These findings complement those of Almuhaya et al. [17], which demonstrated the feasibility of structured ACLR education via Zoom. The asynchronous format used in this work likely reduced participation barriers and contributed to higher completion. Platform-based delivery introduces privacy concerns when clinical communication occurs through channels designed for personal use [46]. These concerns were mitigated here through informed consent and exclusive use of private messaging. The use of an active control group receiving general content through the same platform further supports the specificity of effects observed, suggesting that platform preference alone does not account for the between-group differences.

### 4.4. Early-Phase Targeting and Control Group Improvements

Kinesiophobia peaks during the first three months following ACLR, when maladaptive cognitions remain amenable to modification [15,16,38], as corroborated by longitudinal phase-based data demonstrating a non-linear kinesiophobia trajectory with the greatest reduction occurring during mid-rehabilitation [16]. Targeting TSK-17 > 37 ensured that the intervention reached individuals at the intersection of temporal vulnerability and clinical need, consistent with stepped-care principles [47]. Substantial improvements in the control group reflect that standard ACLR rehabilitation provides graded exposure and reinforces positive recovery expectations; these gains may partly reflect spontaneous cognitive reappraisal processes [6,14]. The intervention group’s larger improvements suggest that structured digital education accelerates and amplifies these natural recovery processes.

**Figure 8:**
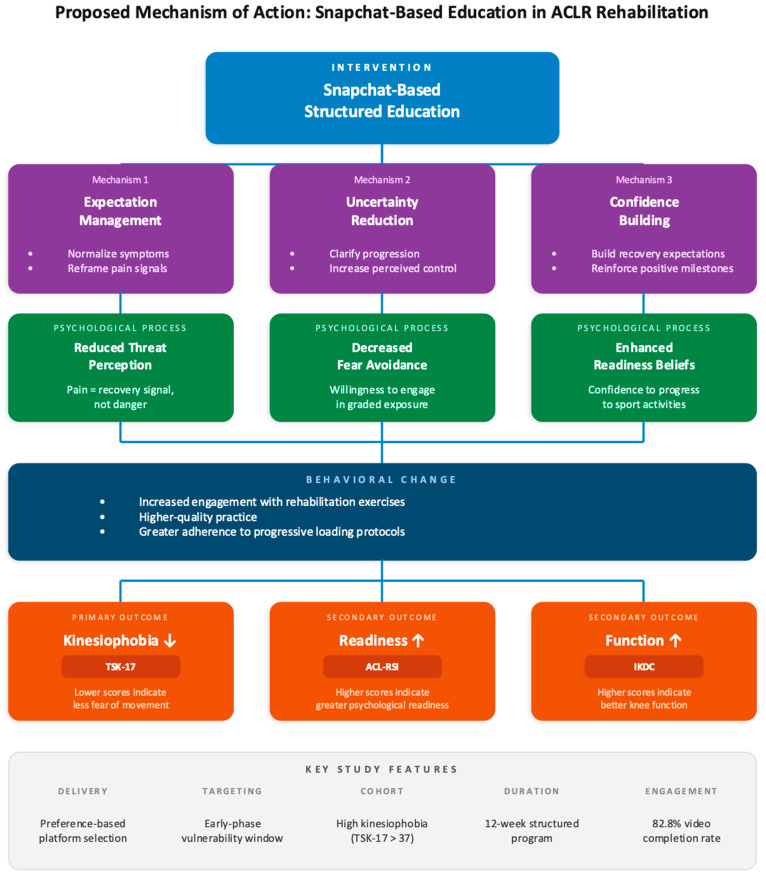
Hypothetical framework for the proposed mechanism of action: Snapchat-based education in ACLR rehabilitation. Note: This figure presents a hypothetical model requiring future empirical validation and is not derived from mediation or biomechanical analyses within the current study. The three intervention mechanisms depicted (expectation management, uncertainty reduction, and confidence building) are theoretical constructs that guided the design of the educational content and were not empirically measured as mediators in the current cohort. Arrows indicate the direction of proposed causal pathways from intervention to mechanisms, psychological processes, behavioral change, and outcomes. The model illustrates how expectation management, uncertainty reduction, and confidence building activate corresponding psychological processes that promote behavioral engagement and produce improvements across all three outcome domains. All between-group differences were statistically significant (*p* ≤ 0.002; η^2^p = 0.068–0.163; *d* = 0.54–0.77).

Improvements in perceived knee function alongside psychological outcomes may operate through several hypothesized pathways: reduced kinesiophobia altering symptom interpretation and self-appraisal, potentially via descending pain modulatory mechanisms [48]; psychological changes facilitating greater engagement with challenging exercises; and synergistic psychological–physical recovery through reciprocal facilitation [49]. Because this study relied exclusively on self-reported outcomes without objective kinematic, biomechanical, or physiological measures, these proposed pathways remain speculative and require empirical validation in future trials that incorporate objective functional assessment and formal mediation analysis (Figure 8).

### 4.5. Clinical Implementation Considerations

Translation of these findings into routine practice requires addressing several implementation challenges. Kinesiophobia screening must be integrated into standard assessment workflows, though adoption faces barriers such as time constraints and uncertainty regarding appropriate referral thresholds [50]. Educational content development requires specialized expertise, suggesting that there is value in disseminating validated programs that clinicians can implement reliably rather than developing original content independently [51]. Digital platform selection must balance patient preferences with privacy protection and organizational policy; commercial platforms evolve rapidly and may introduce changes affecting delivery, whereas dedicated health platforms offer greater control but may sacrifice engagement [52]. Finally, cultural adaptation extends beyond translation to encompass health beliefs, social norms, and communication preferences, requiring formative research to ensure that messaging resonates with local values in each implementation context [53].

### 4.6. Strengths and Limitations

The strengths of this study include validated outcome measures, targeted participant selection, assessor blinding, multiple analytical approaches providing convergent evidence, and high engagement rates validating the feasibility of intervention. The use of an active control (delivering general ACLR information through the same platform and at the same frequency) reduces the likelihood that the observed effects reflect non-specific attention or platform novelty and strengthens the attribution of differences to the structured educational content specifically.

The limitations of this study constrain causal inference. The quasi-experimental design with cluster-level (branch) allocation limits causal attribution despite baseline equivalence. Individual (within-site) randomization was not operationally feasible in this setting for three reasons: First, the intervention was delivered via Snapchat, a peer-connected social platform on which users routinely forward, screenshot, and re-share content; within-site individual allocation would have permitted control participants to be exposed to intervention videos through patient-to-patient sharing, a platform-specific contamination vector that conventional blinding procedures cannot eliminate. Second, branch-level allocation was required to preserve therapist blinding, as within-site randomization would have required the same therapists to treat both arms, making differential verbal reinforcement and unconscious adjustments to in-session counselling difficult to control. Third, participants attending the same branch share waiting areas and rehabilitation gyms, making visual exposure to intervention content on other participants’ mobile devices likely. While the two participating branches operate under identical protocols, staffing models, and training procedures (approximating a matched-site design), the small number of clusters (k = 2) precludes definitive separation of the observed between-group differences from potential branch-level confounding factors, such as unmeasured variation in clinic environment, therapist–patient dynamics, or local cultural factors. This design also precludes reliable estimation of intra-cluster correlation or the use of cluster-robust standard errors; consequently, the a priori power analysis (Section 2.6) was conducted at the individual level. Observed between-group effect sizes (*d* = 0.54 to 0.77) exceeded the planning assumption (*d* = 0.56) for one of three outcomes, providing empirical evidence of adequate signal detection, though the precise contribution of any cluster effect to the observed effects cannot be quantified.

The reported effect estimates should therefore be interpreted as reflecting the combined contribution of the structured educational intervention and any residual cluster-level differences that could not be statistically partitioned. Beyond the design, several additional limitations warrant consideration. Targeting TSK-17 > 37 increases the risk of regression-to-the-mean, potentially inflating within-group change. Exclusive reliance on self-reported outcomes introduces a risk of response bias despite participant blinding, and unmeasured confounders related to clinic culture or clinician characteristics cannot be excluded. Objective functional measures (e.g., limb symmetry index, quadriceps strength, hop tests) were not included because participants were enrolled across a heterogeneous post-operative window (0 to 3 months), and such measures are strongly time-dependent after ACLR; future trials enrolling at a uniform post-operative timepoint should incorporate these measures alongside patient-reported outcome measures (PROMs). In addition, basic objective clinical milestones that are already embedded in our phase-based progression criteria, such as achievement of full knee extension or resolution of effusion, were not extracted from the electronic medical records for secondary analysis in the present work. Retrospective extraction of such phase-appropriate clinical markers represents a valuable direction for future work, as these indicators are less confounded by post-operative timing than performance-based measures and could complement patient-reported outcomes in characterizing recovery.

The 12-week follow-up precludes assessment of long-term RTS outcomes, and generalizability is constrained to Saudi ACLR patients with elevated kinesiophobia who use Snapchat. Among intervention participants lost to follow-up, high video engagement at the time of dropout (mean 10.5 videos viewed) suggests that attrition was unlikely driven by disengagement from the intervention. Furthermore, baseline characteristics of dropouts were comparable to completers across all three outcomes, reducing the likelihood of systematic bias introduced by missing data.

### 4.7. Future Research Directions

Priorities include randomized controlled trials with individual-level randomization and extended follow-up (12–24 months) assessing RTS status, re-injury rates, and objective functional performance; formal mediation analysis to clarify causal pathways linking education to outcomes; dose–response investigations examining engagement–outcome relationships; platform comparison studies delivering identical content across multiple channels to isolate platform-specific effects; and implementation research addressing cost-effectiveness and dissemination across diverse clinical settings.

## 5. Conclusions

This study provides evidence that structured educational content delivered through a patient-preferred digital platform, as an adjunct to standard ACLR rehabilitation, is associated with statistically significant improvements in kinesiophobia, psychological readiness, and perceived knee function, with effect sizes ranging from moderate to moderate-to-large, although changes on TSK-17 and ACL-RSI did not definitively exceed established MCID or SDC thresholds. High engagement rates validate preference-based platform selection as a critical design principle in digital health development, and the consistency of findings across both per-protocol and intent-to-treat analyses strengthens confidence in intervention effects despite the quasi-experimental design. Clinical implications include routine kinesiophobia screening to identify high-risk patients, implementation of structured digital education as a scalable adjunct to standard care, and prioritization of patient preference in platform selection.

## Figures and Tables

**Table 1:** Baseline demographic, clinical, and outcome characteristics.

Variable	Control (*n* = 60)	Intervention (*n* = 60)	Test Statistic	*p*
Demographic Characteristics (Mean ± SD)				
Age (years)	28.55 ± 7.11	27.95 ± 6.83	*t* = 0.47	0.638
Height (cm)	170.62 ± 8.61	170.20 ± 9.42	*t* = 0.25	0.801
Weight (kg)	74.08 ± 11.98	74.65 ± 10.57	*t* = −0.28	0.784
BMI (kg/m^2^)	25.80 ± 4.28	26.23 ± 4.56	*t* = −0.53	0.596
Clinical Characteristics				
Rehabilitation Phase [*n* (%)]			χ^2^ = 2.19	0.139
Phase 1	29 (48.3)	21 (35.0)		
Phase 2	31 (51.7)	39 (65.0)		
Physical Therapy Sessions (Mean ± SD)	23.17 ± 3.60	23.43 ± 3.72	*t* = −0.40	0.691
Graft Type [*n* (%)]			χ^2^ = 0.78	0.855
Patellar Tendon	23 (38.3)	20 (33.3)		
Hamstring Tendon	13 (21.7)	17 (28.3)		
Quadriceps Tendon	15 (25.0)	14 (23.3)		
Allograft	9 (15.0)	9 (15.0)		
Dominant Leg (Right) [*n* (%)]	34 (56.7)	25 (41.7)	χ^2^ = 2.70	0.100
Injured Leg (Right) [*n* (%)]	35 (58.3)	31 (51.7)	χ^2^ = 0.54	0.463
Baseline Outcome Scores (Mean ± SD)				
TSK-17	43.40 ± 4.34	42.92 ± 4.29	*t* = 0.61	0.541
ACL-RSI	40.68 ± 5.41	39.25 ± 5.36	*t* = 1.46	0.148
IKDC	47.46 ± 12.10	48.40 ± 12.75	*t* = −0.41	0.681

Note: TSK-17 = Tampa Scale for Kinesiophobia (17–68; higher = greater kinesiophobia); ACL-RSI = ACL Return to Sport after Injury (0–100; higher = greater readiness); IKDC = International Knee Documentation Committee Subjective Knee Form (0–100; higher = better function).

**Table 2:** Descriptive statistics at baseline and 12 weeks (per-protocol completers).

Outcome	Control (*n* = 52)			Intervention (*n* = 50)		
	Baseline	Week 12	Δ	Baseline	Week 12	Δ
TSK-17	43.96 ± 3.96	42.13 ± 5.55	−1.83	43.00 ± 4.27	38.50 ± 4.82	−4.50
ACL-RSI	40.98 ± 5.40	54.73 ± 10.78	+13.75	39.10 ± 5.53	61.36 ± 10.19	+22.26
IKDC	46.48 ± 12.23	58.83 ± 15.34	+12.35	48.09 ± 13.31	68.66 ± 16.90	+20.57

Note: Δ = mean change from baseline to 12 weeks. Baseline values reflect per-protocol completers only.

**Table 3:** Repeated measures ANOVA: time × group interaction effects (per-protocol completers).

Outcome	RM-ANOVA		
	Time × Group *F*(1,100)	*p*	η^2^p
TSK-17	11.78	<0.001	0.105
ACL-RSI	19.54	<0.001	0.163
IKDC	7.317	0.008	0.068

Note: TSK-17 = Tampa Scale for Kinesiophobia; ACL-RSI = ACL Return to Sport after Injury scale; IKDC = International Knee Documentation Committee Subjective Knee Form. η^2^p = partial eta squared.

**Table 4:** ANCOVA: Baseline-adjusted between-group comparisons at week 12 (per-protocol completers).

Outcome	Control Adjusted Mean	Intervention Adjusted Mean	Mean Difference	95% CI	*F*(1,99)	*p*	η^2^p	d
TSK-17	41.74	38.91	−2.82	[−4.37, −1.28]	13.19	<0.001	0.118	0.54
ACL-RSI	54.03	62.09	+8.06	[4.18, 11.95]	17.17	<0.001	0.148	0.77
IKDC	59.29	68.19	+8.90	[3.23, 14.57]	9.670	0.002	0.089	0.54

Note: Mean differences are expressed as intervention minus control. Positive values for ACL-RSI and IKDC favor intervention; negative values for TSK-17 favor intervention.

**Table 5:** Primary intent-to-treat analysis using Multiple Imputation (M = 50): pooled ANCOVA baseline-adjusted between-group comparisons at week 12 (*n* = 120).

Outcome	Adjusted Mean Difference	SE	95% CI	t	df	*p*	Cohen’s d	FMI
TSK-17	−2.94	0.75	[−4.43, −1.45]	−3.90	101.4	<0.001	0.75	0.10
ACL-RSI	+7.93	1.95	[+4.07, +11.80]	+4.08	93.2	<0.001	0.82	0.15
IKDC	+8.85	2.88	[+3.13, +14.56]	+3.07	93.7	0.003	0.61	0.15

Note: Fifty imputed datasets were generated via MICE (multivariate imputation by chained equations), with 20 burn-in iterations; ANCOVA models were fitted within each and pooled using Rubin’s rules with Barnard–Rubin adjusted degrees of freedom. Mean differences are expressed as intervention minus control; positive values for ACL-RSI and IKDC favor intervention, and negative values for TSK-17 favor intervention. FMI = fraction of missing information.

**Table 6:** Sensitivity analysis using Last Observation Carried Forward (LOCF) repeated measures ANOVA: time × group interaction effects (*n* = 120).

Outcome	RM-ANOVA		
	Time × Group *F*(1,118)	*p*	η^2^p
TSK-17	9.563	0.002	0.075
ACL-RSI	10.46	0.002	0.081
IKDC	5.218	0.024	0.042

Note: LOCF-based sensitivity analysis included all 120 enrolled participants. Results are convergent with the primary Multiple Imputation ITT analysis (Table 5). η^2^p = partial eta squared.

## Data Availability

The data supporting the findings of this study are not publicly available due to ethical and privacy restrictions related to patient confidentiality. De-identified data may be made available from the corresponding author upon reasonable request and subject to institutional approval.

## Abbreviations

The following abbreviations are used in this manuscript:

ACLR	Anterior Cruciate Ligament Reconstruction
ACL-RSI	ACL Return to Sport after Injury Scale
ANCOVA	Analysis of Covariance
ANOVA	Analysis of Variance
BMI	Body Mass Index
CI	Confidence Interval
FMI	Fraction of Missing Information
ICC	Intraclass Correlation Coefficient
IKDC	International Knee Documentation Committee Subjective Knee Form
ITT	Intent-to-Treat
LOCF	Last Observation Carried Forward
MCID	Minimal Clinically Important Difference
RTS	Return to Sport
SD	Standard Deviation
SDC	Smallest Detectable Change
SEM	Standard Error of Measurement
TSK-17	Tampa Scale for Kinesiophobia (17-item version)
